# Analysis of Mechanical Properties and Parameter Dependency of Novel, Doubly Re-Entrant Auxetic Honeycomb Structures

**DOI:** 10.3390/polym16172524

**Published:** 2024-09-05

**Authors:** Levente Széles, Richárd Horváth, Lívia Cveticanin

**Affiliations:** 1 Doctoral School on Materials Sciences and Technologies, Óbuda University, H-1034 Budapest, Hungary; szeles.levente@cl.uni-obuda.hu; 2 Bánki Donát Faculty of Mechanical and Safety Engineering, Óbuda University, H-1034 Budapest, Hungary; 3 Faculty of Technical Sciences, University of Novi Sad, 21000 Novi Sad, Serbia; cveticanin@uns.ac.rs

**Keywords:** auxetic metamaterial, additive manufacturing, compression testing, novel unit-cell design, parameter dependency, finite element modelling

## Abstract

This study proposes a new, doubly re-entrant auxetic unit-cell design that is based on the widely used auxetic honeycomb structure. Our objective was to develop a structure that preserves and enhances the advantages of the auxetic honeycomb while eliminating all negative aspects. The doubly re-entrant geometry design aims to enhance the mechanical properties, while eliminating the buckling deformation characteristic of the re-entrant deformation mechanism. The effects of the geometric modification are described and evaluated using two parameters, offset and deg. A series of experiments were conducted on a wide range of parameters based on these two parameters. Specimens were printed via the vat photopolymerization process and were subjected to a compression test. Our aim was to investigate the mechanical properties (energy absorption and compressive force) and the deformation behaviour of these specimens in relation to the relevant parameters. The novel geometry achieved the intended properties, outperforming the original auxetic honeycomb structure. Increasing the *offset* and *deg* parameters results in increasing the energy absorption capability (up to 767%) and the maximum compressive force (up to 17 times). The right parameter choice eliminates buckling and results in continuous auxetic behaviour. Finally, the parameter dependency of the deformation behaviour was predicted by analytical approximation as well.

## 1. Introduction

**Figure 1:**
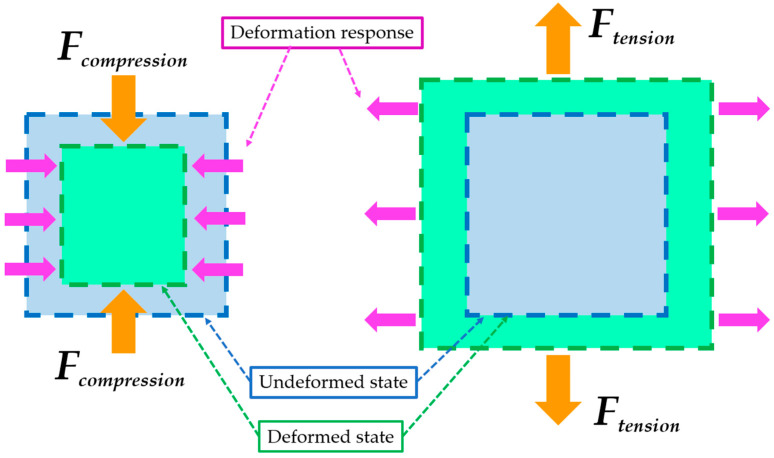
Illustration of the auxetic behaviour for both compressive and tensile loads; based on [24].

Most engineering materials expand when compressed. The relationship between transverse and longitudinal strain is described by Poisson’s ratio, which is typically between 0 and +0.5 [1,2]. However, certain materials shrink laterally when compressed and expand under tensile load, and these materials have a negative Poisson’s ratio (this effect is illustrated in Figure 1). 

Materials with a negative Poisson’s ratio were named auxetic materials by Evans et al. [3]. The history of auxetic materials dates back almost 100 years, to 1927–1928 [4]. Poisson’s ratio of the traditional honeycomb lattice was derived by Gibson et al. [5] in their 1982 publication. The formula was also plotted for negative values of Poisson’s ratio. The advantages of auxetic materials include higher energy absorption [6,7], increased hardness [8], indentation resistance [9], high-speed impact resistance (i.e., blast resistance) [10], and increased fracture resistance [11,12]. These properties are effectively applied in automotive crumple zones, e.g., bumpers [13], airless vehicle tyres [14], sport helmets [15], and seismic dampers in civil engineering [16], and it is important to mention the application of such structures in the military [17]. In civil engineering, auxetic foams are used for filtration [18] and are also mixed into mortars, with renders and the concrete serving as protective layers [19,20]. Two significant periods can be highlighted in the history of auxetic materials. The first one is the second half of the 1980s, when researchers analysed various geometrical and topological effects and essentially made the auxetic behaviour designable [21,22,23].

**Figure 2:**
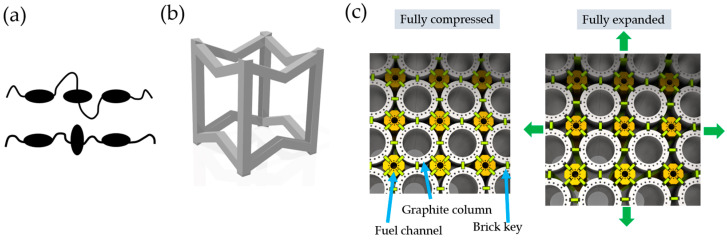
Examples of auxetic structures at different scales. (**a**) On a molecular level; auxetic trimer in nematic and stretched phase (based on [35]); (**b**) auxetic unit cell (which is a few millimetres in size), designed with additive manufacturing in mind (based on [36]); (**c**) large-scale auxetics in a nuclear power plant (based on [37]). Owing to the auxetic design, the reactor core can withstand earthquakes and has a high resistance to horizontal shear deformation, which was a basic requirement for these types of nuclear power plants.

The topic of auxetics gained more serious attention in the 1990s with a plethora of real-life applications. In 1991, Choi et al. [25] created a smart rived based on auxetic foams. Evans et al. [4] stated that auxetic seals create a more secure seal. Auxetic materials are not only found in manmade structures, but we are also surrounded by many substances in nature that exhibit auxetic behaviour. The udder of cows [26] and the skin of cats [27] and salamanders [28] also show auxetic behaviour. Furthermore, 69% of cubic elemental metals exhibit a negative Poisson’s ratio in the {110} direction [29]. Other auxetic materials with a negative Poisson’s ratio include alpha-Cristobalite [30]; single-crystalline arsenic, antimony, and bismuth [31]; and cadmium and thallium [32]. Researchers also take inspiration from nature in hopes of improving manmade auxetic structures [33]. Researchers have also become interested in polymers and have synthesised a new class of auxetic polymers by molecular engineering [34] and investigated their behaviour [35]—see Figure 2a. The auxetic behaviour is limitless and scaleless. Figure 2 illustrates the scalability of auxetics; Figure 2b shows an example of an auxetic unit cell, which is a few millimetres in size and made with additive manufacturing technologies [36]; while Figure 2c shows the large scale auxetic “graphite core” of many nuclear reactors opened in the 1950s [37]. Auxetic structures are now created and investigated even in the nanometric scale [38]. The limitlessness of auxetics allows for the creation of metamaterials from various materials, such as foams [39] and ceramics [40].

**Figure 3:**
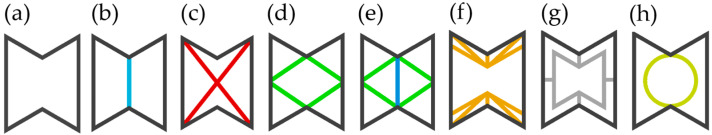
Examples of improved auxetic honeycomb unit cells. (**a**) The original auxetic honeycomb; (**b**) embedded horizontal rod (based on [46]); (**c**) transversely connected corners (based on [47]); (**d**) embedded rectangular stiffening (based on [48]); (**e**) the combination of figures (**b**,**d**) (based on [49]); (**f**) complex geometrical stiffening (based on [50]); (**g**) self-repeating “offset” stiffening (based on [51]); (**h**) circular reinforcement (based on [52]).

The second period full of breakthroughs was really brought about by the emergence of additive manufacturing (AM) technologies and the designability of these structures; however, auxetic lattices with simple geometries can be created using conventional manufacturing techniques [41,42]. More complex lattice structures can be manufactured using conventional manufacturing techniques and ingenuity. Yilin et al. [43] proposed the application of the mortise and tenon principle to demolish the bottleneck of relying solely on AM for production. Undoubtedly, the emergence of new technologies [44,45] increased the feasibility of auxetic structures in real-life applications. The negative Poisson’s ratio of manmade auxetic structures is achieved by clever geometric designs. There are several deformation mechanisms. For example, the auxetic honeycomb (Figure 3a) is classified in the group of re-entrant mechanisms [7]. In such structures, re-entrant edges “open” under a tensile load, thus expanding the structure, while the opposite behaviour is observed under a compressive load. 

The nested geometry approach is the most common way to enhance the properties of the auxetic honeycomb structure. Lu et al. [46], in 2016, increased the elastic modulus of the structure by incorporating a horizontal rod, as shown in Figure 3b. Transversely connecting the corners of the unit cell by two bar elements (Figure 3c) increases the in-plane stiffness and Young’s modulus value [47]. The authors also considered another stiffening arrangement, shown in Figure 3d, to be effective [48]. The presented modification by Lu et al. [46] and Fu et al. [48] were combined by Chen et al. [49] (Figure 3e). Self-similar inclusion is another widely researched approach which can significantly increase the stability of the auxetic honeycomb structure [50,51,53] (see Figure 3f–g). Yilin et al. [54] replaced the re-entrant edges of the unit cell with “zig-zag” elements, thus increasing the stiffness and conjugate auxeticity of the structure. In addition to the traditional straight rod element-based structures, partly rounded [55] and curved geometric designs and modifications also received considerable attention [56]. Rounded and curved embedded segments introduce grater overlapping sections, hence increasing the stiffness, energy absorption [57], and auxeticity of the structure [58], while eliminating the effect of stress concentration in sharp corners [59]. Ehsan et al. [57] were even able to create an auxetic honeycomb structure made up of arcs. The deformation mechanism changed from bending dominated to stretching dominated, allowing the researchers to obtain tuneable and even constant Poisson’s ratios. Curved segments can also improve the properties of the auxetic honeycomb unit cell as well (see Figure 3h) [52,60]. Nesting can improve mechanical properties for 3-dimensional lattices as well [61]. 

The need for improvement stems from the structural motifs of the re-entrant mechanism. Re-entrant structures have auxeticity for both compression and tensions, thus making them more widely applicable compared to other auxetic structures. Re-entrant structures reflect most of the favourable auxetic properties, and their low porosity makes them useful for lightweight designs. Unfortunately, their thin ribs are prone to flexure and shear deformation, posing an obstacle to auxeticity, as buckling may occur [7]. Once buckling occurs, we are no longer able to harvest all the advantageous properties of the structure. 

Another group of deformation mechanisms mentioned earlier is the “chiral” mechanism, which has a different behaviour mechanism than “re-entrant” structures [7]. However, the individual mechanisms can be combined. Reza et al. [62]. Wei. et al. [63] acknowledged the buckling tendency of the auxetic arrowhead honeycomb and incorporated that into their novel structure. In their work, they created an improved double-arrow honeycomb structure with a two-step deformation mode. First, they allowed buckling to happen, and then, with their geometrical modification, auxetic shrinkage took place.

Lattice structures are usually considered as part of a regular pattern, a pattern made up solely of neighbouring unit cells with shared edges; however, the unique properties of auxetic lattices can be harvested, even if they do not form a structured grid. Ali et al. [64] and Ramen et al. [65] investigated the stiff re-entrant honeycomb structure as a free-standing (i.e., not latticed) unit cell wrapped around a cylinder and as a 2.5-dimensional structure, respectively; this unit-cell design was a major inspiration for our novel unit cell. This novel stiff unit cell introduces one breakpoint at each re-entrant edge and connects the two original ones with a horizonal edge. The stiffened structure outperformed the original, soft structure in both papers; thus, this geometrical modification can be considered as an effective way of stiffening. Furthermore, a graded layout (i.e., including both soft and stiff honeycombs) leads to quasi-zero stiffness under compression, making these structures superior vibration isolators [64]. In these studies, the free-standing configuration led to buckling and non-uniform, unpredictable deformation behaviour, and high-performance lattice structures need to be predicable. The newly added centre horizontal edge would hinder lattice-like behaviour, if these unit cells were to be arrayed as neighbouring unit cells; furthermore, the parameter dependency of these newly added breakpoints on the re-entrant edges would be of great interest. In the case of other novel auxetic structures, mechanical properties are parameter-dependent, such as the equivalent elastic modulus, which is closely related to the re-entrant angle [66].

The novel or modified structures presented above are 2.5-dimensional; however, novel structures were not only created in 2.5 dimensions. An excellent intermediate structure is the novel auxetic structure created by Dong et al. [67]. Their structure is 3-dimensional, based on a 2.5-dimensional structure bent around a cube. These structures are also referred to as tubular structures, which have gained considerable popularity recently, and the tubular auxetic design was also combined with the gradient design with hopes of biomedical applications [68]. 

Hybrid structures were also created in 3 dimensions as well by Qifang et al. [69]; in their research, they compared hybrid and basic structures where the outer and inner frame were made of auxetic or non-auxetic honeycomb lattices. Examining all four possible variations, they found that hybrid structures showed greater stiffness values [69]. Multilateral stiffening has also received considerable attention lately [70,71]. Another aspect of multilateral stiffening is when traditional building elements are stiffened by lattice filling [72]. Lastly, it is crucial to mention the thickness gradient design as a method for improving mechanical properties [73,74]. The gradient design can be considered as the parent idea for a variety of geometrically constrained designs, in which researchers enhance certain regions of a specimen in hopes of improving mechanical parameters with great success [75]. 

Finally, it is important to develop cost-effective, fast, and highly accurate lattices; thus, a well-established finite element environment is key, similar to technical creations [76], construction problems [77,78], or thermoelectric problems [79], for example.

By evaluating the methods presented in this literature review, we began our own development. Our objective was to retain all beneficial properties of the re-entrant mechanism while eliminating all negative aspects. To retain the re-entrant mechanism, we proposed the doubly re-entrant structure in hopes of eliminating buckling behaviour and enhancing mechanical properties (such as compressive resistance). 

In addition to geometric improvements, we examine the impacts of different geometric parameters in this publication. Using the proper manufacturing technology and material enabled an examination of the geometry over a wide deformation range. 

This paper is divided into four sections. After the Introduction in Section 2, the stiff novel, doubly re-entrant auxetic unit cell and the investigated parameters are introduced. The specimen behaviour is investigated by theoretical approximation, finite element simulations, and real compression tests; hence, the test environment for each method is described. Specimens are fabricated using additive manufacturing, and the manufacturing parameters and material properties are also presented in Section 2. Section 2 ends with the presentation of theoretically approximated mechanical parameter values and a presentation of the expected deformation behaviour. Section 3 presents and compares the results of the three test methods. Specimens are characterized by their energy absorption, specific energy absorption, maximum compressive force, and deformation behaviour. A strong parameter dependency can be obtained for each property. Each investigated parameter combination surpasses the original re-entrant honeycomb unit cell, which also formed the scope of our study. This paper ends with Conclusions in Section 5. 

## 2. Materials and Methods

In this section, the generation and geometrical parametrization of our novel unit cell is introduced. The novel unit cell is overlayed over the original re-entrant honeycomb unit cell for comparison and illustration. The geometrical modification is characterized by two geometrical parameters, the effects of which were investigated. Once the unit cell is introduced, specimens made up from these unit cells in a neighbouring lattice pattern are shown, alongside all the investigated parameter combinations. The introduction of the novel geometry is followed by a description of the manufacturing procedure and base material testing procedure. Mechanical parameters and the deformation behaviour form the foci of our result evaluation, and these parameters were obtained by compression testing. Compression testing was carried out using compression-testing equipment and simulations using the finite element method (FEM). Testing parameters and FEM calculation details are also presented in this section. The expected deformation behaviour and mechanical properties, such as Poisson’s ratio, are predicted by a complex analytical approach.

### 2.1. Introducing the Novel Geometry

The auxetic honeycomb has a re-entrant deformation mechanism; thus, when a compressive load is applied, the re-entrant edges deform inward. Owing to the re-entrant deformation mechanism, the structure has auxeticity not only for tensile but for compressive loads as well, making them widely applicable [8]. The aim was to develop a novel structure that preserves the advantages of this deformation mechanism and the structure while eliminating the disadvantages. 

In the case of the buckling of re-entrant structures, flexure and shear deformations are typical [8]. Furthermore, the resistance to the deformation load of the structure is low. As investigated in a recent study, compressive parameters can be improved with proper material selection, but the base material has little to no effect on the negative (buckling) deformation behaviour of the structure [80]. Given the foregoing, we aimed to give the geometry more favourable properties by creating a doubly re-entrant structure. 

**Figure 4:**
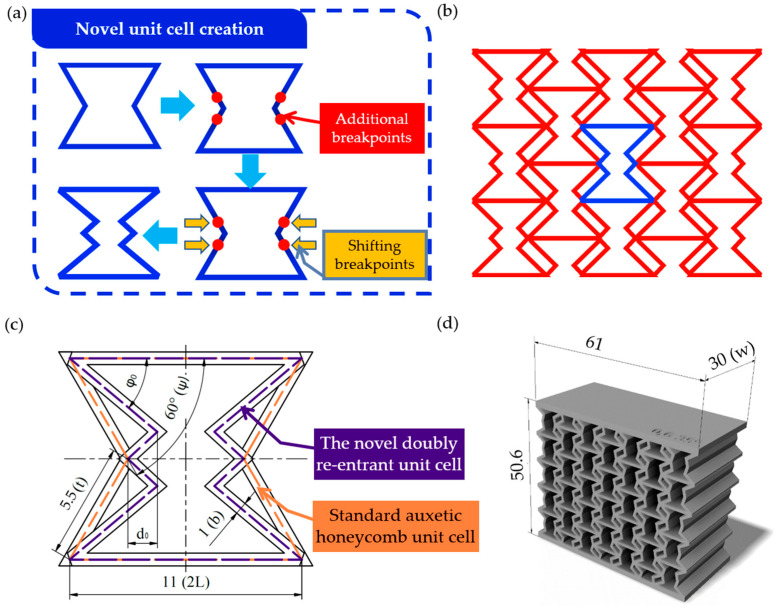
Illustration of creating the novel, doubly re-entrant unit cell and forming a specimen from these unit cells. (**a**) The process of creating the novel, doubly re-entrant unit cell in 4 steps by introducing a breakpoint (red dots) at each re-entrant edge of the original re-entrant honeycomb unit cell; (**b**) an illustrative pattern of the novel unit cells, arranged into a lattice as neighbouring unit cells with shared edges; (**c**) the main dimensions of the novel unit cell and the two parameters used to describe the geometrical modification (namely, the offset (offset—$d_{0}$) and angle (deg—$\varphi_{0}$)); (**d**) enclosing dimensions of specimens to be tested. All dimensions shown in this figure are in millimetres.

As illustrated in Figure 4a, the re-entrant edges of the auxetic honeycomb unit cell were provided with a breakpoint, which shifted towards the centre of the unit cell. Figure 4b illustrates a lattice made up of these unit cells. As a result of the broken edges, unlike in the original structure, the re-entrant edges do not overlap with each other. A rectangular section is formed between the modified edges, which is not auxetic. The doubly re-entrant geometry can, in theory, improve the auxeticity of the structure, and long parallel edges of the resulting rectangular sections can increase the stability of the structure. It must be noted that compared to the original structure, as the re-entrant edges do not overlap, the modified unit cell consists of more edges; thus, the results must also be evaluated by weight. 

### 2.2. Geometrical Description of the Tested Specimens

The novel unit cell was characterized by two parameters, the offset (*offset—*$d_{0}$) and angle (*deg*—$\varphi_{0}$) parameters, shown on Figure 4c. The *offset* parameter is the amount of shift (displacement) of the breakpoints compared to the breakpoints of the original structure. The *deg* angle value is the angle enclosed by the horizontal and the upper re-entrant edges. The *offset* and *deg* parameters are measured from the centrelines of the unit cells, which are indicated by dashed lines in Figure 4c. The overall dimensions and notations of the unit cell are also presented in Figure 4c. Specimens are assembled from a regular pattern of these unit cells. Specimens must contain enough unit cells to reliably characterize their behaviour. Thus, the specimens were made up of 5-by-7 unit cells, and in order to avoid out-of-plane buckling, the specimens were 30 mm deep (dimension *w*; see Figure 4d). The enclosing dimensions of the specimens are shown in Figure 4d. A solid sheet with a thickness of 2 mm, responsible for transferring the load during compression testing, was formed on the upper and lower part of the specimens. To evaluate the effects of the geometric modification, a series of experiments were created in which each specimen was significantly different, each with its own initial parameter data listed in Table 1. Relative density values were calculated using CAD data, and density values were calculated by multiplying the obtained relative density values with that of the forming material (see Section 2.4). A representation of the CAD model for each parameter combination is attached in Appendix A. 

In addition to the series of experiments consisting of 9 specimens, a sample specimen (referred to as “*Etalon*”) built from auxetic honeycomb unit cells was also printed and then studied for comparability. 

### 2.3. Preparation of Specimens

Specimens were made by the vat photopolymerization additive manufacturing process, specifically by masked stereolithography (mSLA) on an Anycubic Photon M3 Printer (Hongkong Anycubic Technology Co., Limited, Hongkong, China). The specimen production was performed with a unique resin mixture that is flexible enough to evaluate deformational behaviour without fractures but also has some degree of load resistance (firm). The added load resistance of the firm component was required for increased the measuring accuracy. 

To achieve this mixture, 37% Photocentric U DLP Firm (grey; 1180 $kg/m^{3}$) and 63% Resione F69 (black; 990 $kg/m^{3}$) elastic resin were homogeneously mixed, and the properties of the raw materials are presented in Section 2.4. Specimens were printed with an exposure time of 4.5 s per layer, with a 0.05 mm layer thickness, at an angle of 14 degrees relative to the build platform, with a 4 mm offset and a dense support structure. Once removed from the build platform, the specimens are soaked in an isopropyl alcohol (99.8% purity) bath for 3 min, followed by a 15 min cycle in the Anycubic Wash & Cure 2.0 25 W (Hongkong Anycubic Technology Co., Limited, Hongkong, China) resin hardener to acquire their final properties.

### 2.4. Mechanical Properties of the Resin Mixture

**Figure 5:**
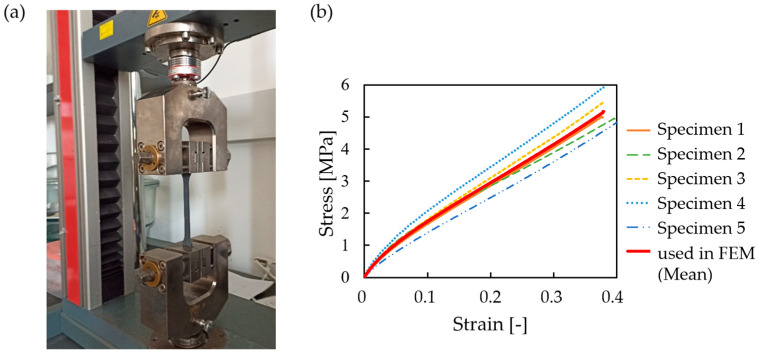
Tensile-testing procedure. (**a**) Tensile-testing specimen during tensile testing using a Zwick Z020-type tensile-testing machine; (**b**) the evaluated tensile test results plotted as stress–strain curves for all 5 specimens and a calculated mean curve, which will be used for the finite element method simulations.

To acquire the mechanical properties of the mixture described in the previous section, a series of tensile tests were conducted on the printed specimens, according to EN ISO 527-2 [81], using a Zwick Z020 (ZwickRoell GmbH & Co. KG, Germany, Ulm)-type tensile-testing machine with a measuring limit of 20 kN. The tensile specimens were prepared with a thickness of 4 mm (as per the standard), and thicker 6 mm versions were made as well, since specimens break at low tensile-force values. Tests were carried out at a speed of 5 mm/min. The tensile test process and the evaluated stress–strain curves appear in Figure 5a and Figure 5b, respectively. Tensile test results deviate only slightly from each other; the mean stress–strain values are used as the input for the finite element material model (see Figure 5b). Based on the graphics, it is evident that the modulus of elasticity for the material is E=13.55 MPa and the stress is $\sigma_{p}=6.12\;MPa$. The density of the resin mixture is 1100 $kg/m^{3}$. 

### 2.5. Compression Test Parameters

Compression tests were conducted to characterize the mechanical properties and deformational behaviour of the specimens. Measurements were conducted on a Hegewald & Peschke (Hegewald & Peschke Meß- und Prüftechnik GmbH, Nossen, Germany) 40-ton-capacity machine at a low speed of 5 mm/min up to 30 mm of deformation. At 30 mm of deformation, the specimens were almost completely compacted. The compression test results are analysed in detail in Section 3.1, but it can be stated at the outset that the specimens exhibited two significantly different behavioural mechanisms during compression. Initially (at low deformation loads), auxetic behaviour was observed in all cases; however, after a certain point, one group of specimens buckled laterally, while the other group exhibited a (preferred) continuous auxetic behaviour. What is more, an analytical description is proposed to explain the phenomenon. 

### 2.6. Finite Element Method Test Environment

The finite element method makes it possible to determine and design the behaviour of a given lattice structure without prior real-life measurements. Naturally, the finite element method requires validation to determine the accuracy of the model. In this study, finite element tests are performed using Ansys Workbench 2023 R2. The finite element test environment was designed to reproduce the real compression test environment, so the moving and stationary jaws of the compression-testing machine are also part of the finite element method (FEM) model.

**Figure 6:**
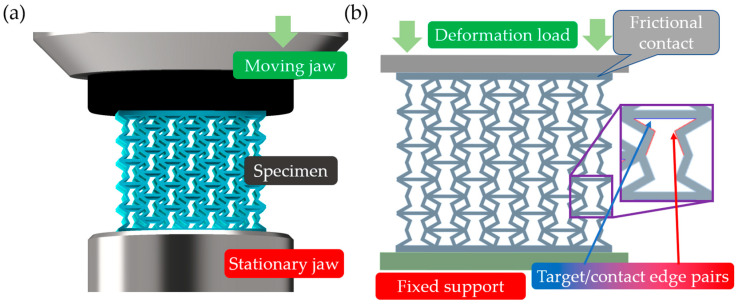
Reproducing the compression-testing procedure in a finite element environment. (**a**) Illustrating the real test environment as a CAD model consisting of a specimen to be tested and the moving and stationary jaws of the tensile-testing machine; (**b**) the simplified FEM representation of the test environment is shown, consisting of the moving and stationary jaws of the machine and a specimen, boundary conditions, and contact pairs are also a part of this figure.

**Figure 7:**
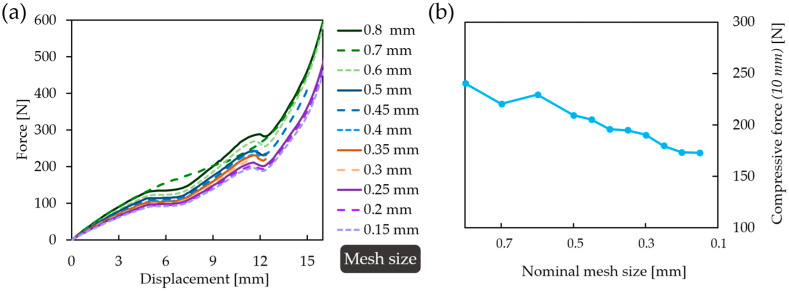
The effect of mesh size on simulation convergence and results. (**a**) Force–displacement curves of a specimen compression obtained at different mesh sizes; (**b**) compressive force measured at 10 mm of deformation next to different mesh sizes.

Considering the experimental compression tests and the dimensions of the specimens, it can be stated that no out-of-plane buckling occurs, so 2-dimensional (plane stress) simulations are adequate. Figure 6a illustrates the real compression test, while Figure 6b illustrates the finite element representation. The load was deformation-controlled and applied in steps (incrementally; 1 mm of deformation equals 1 step) to the upper edge of the moving jaw. In accordance with the real compression tests, the stationary lower jaw was held rigidly. The large deformation option was turned on for the specimen. The coefficient of friction between the specimen and the stationary and moving jaws of the testing machine was 0.2, while the coefficient of friction between the potentially contacting edges of the specimen was set to 0.35 [82]. The contact detection method was set to nodal-normal to target to achieve convergence at large deformations. The mesh size varied for the specimen and the jaws, and a 0.8 mm element size was chosen for the moving and stationary jaws. Regarding the non-linear nature of the problem, a mesh convergence study was carried out to determine the appropriate mesh size for the specimens. Figure 7a illustrates the force–displacement curves obtained next to the different mesh sizes; decreasing the mesh size resulted in convergence at increasing deformation loads. Figure 7b shows the maximum force value at a 10 mm displacement next to the different mesh sizes; one can see that decreasing the mesh size below 0.2 mm is not required, based on Figure 7b. The required mesh size for extended convergence should be at least 0.25 mm. A 0.2 mm mesh size was chosen for specimen meshing.

The quadrilateral dominant meshing method with a linear element order was used to achieve more stable convergence at large strain values. As the deformation progresses, the specimen and the mesh are significantly distorted; therefore, an adaptive remeshing module was applied to the specimen. A hyperplastic two-parameter Mooney–Rivlin model (originally derived by Mooney [83] and expressed in terms of the Cauchy–Green deformation tensor invariants by Rivlin [84]) was fitted in the finite element software to the stress–strain values determined from the tensile test results described in Section 2.4. Using the established finite element environment, the scope of our investigation can be expanded in the future without the need to produce more specimens. 

### 2.7. Analytical Approach

In this subsection, an analytical approximation method is used to describe the deformation behaviour and mechanical properties of the novel unit cell.

#### 2.7.1. Description of the Continuous Auxetic Deformation Behaviour

**Figure 8:**
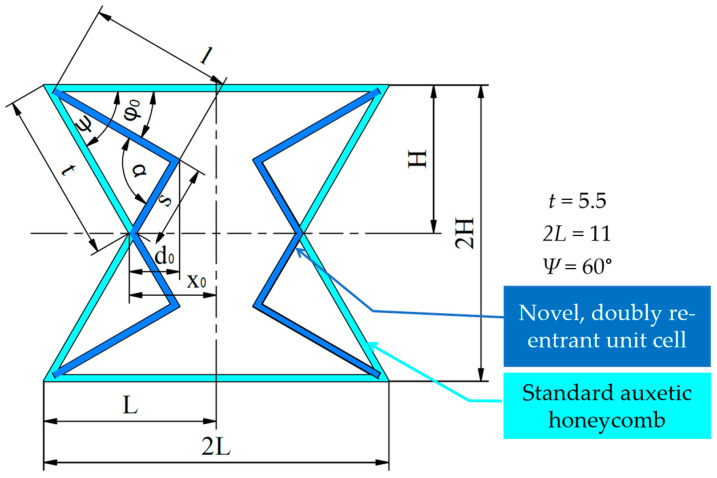
Notations and dimensions of the unit cell used for the analytical method. The unit cell is illustrated as an overlay to the original re-entrant honeycomb unit cell at the initial, undeformed stage.

The variables used in the equations for the analytical approach are according to the notations in Figure 8. Based on Figure 4c and Figure 8, the known dimensions are t=5.5 mm, 2L=11 mm, and ψ=60°. The offset and deg parameters introduced in Figure 4c are denoted by d and φ, respectively. As the deformation progresses, their values change as well. The initial values are distinguished by $d_{0}$ and $\varphi_{0}$.

The unit-cell geometry is described by the equations attached in Appendix B. These equations form the basis for the analytical approximation.

#### 2.7.2. Deformation of the Unit Cell and Characterization of Poisson’s Ratio

**Figure 9:**
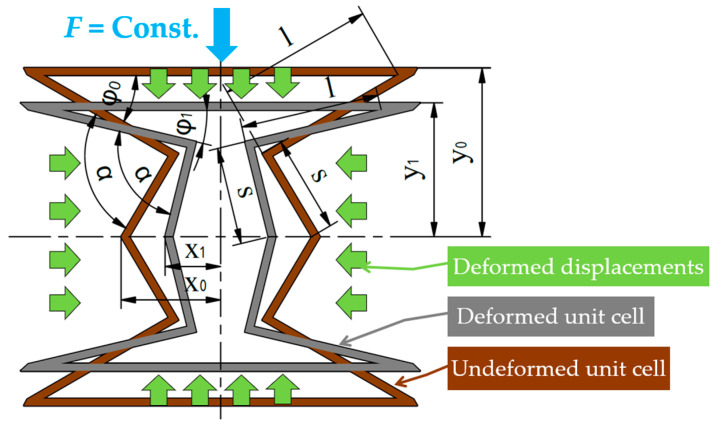
Deformation of the unit cell and its main dimensions (changing dimensions are indexed, where index ”0” refers to the initial stage, and index ”1” refers to a representative deformed stage). Deformation is induced by a compressive vertical force, as shown in this figure.

Deformation occurs due to a constant vertical (*F* = *Const*) force acting on the upper surface of the cells. The height (*y*) of the unit cell decreases, and the width (*x*) contracts (see Figure 9).

Deformational changes in the *x* and *y* directions, occurring due to the load, can be described by the two parameters ($d_{0}$ and $\varphi_{0}$) used for characterising the geometrical modification as follows:

(1) $$y=lsin\varphi +\sqrt{s^{2}-d^{2}},$$

(2) x=L−lcosφ+d.

Let us introduce the relation for the offset–generalized form of Equation (A5):

(3) d=s cos(α−φ).

Eliminating φ in A6 and using (1) (2), the *y*–*x* relation is follows as:

(4) $$y=\sqrt{l^{2}+s^{2}-2ls\cos\alpha -{\left(L-x\right)}^{2}}.$$

**Figure 10:**
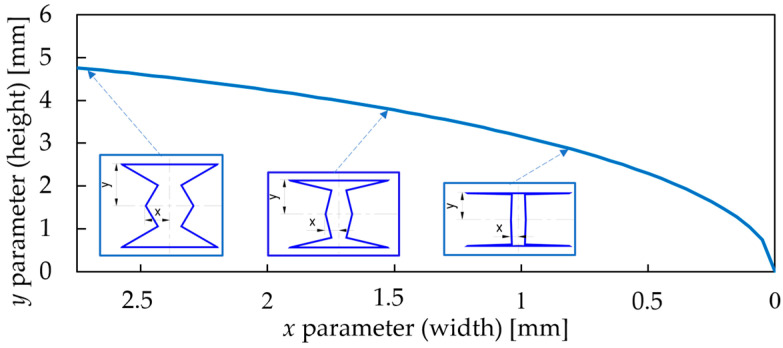
The relationship of the x–y dimension as the deformation progresses for the novel unit cell. Values are plotted, starting from the initial, undeformed unit-cell dimensions for a unit cell characterized by $\varphi_{0}$ = 30° and $d_{0}=1.4\;mm$.

Figure 10 illustrates the *x*–*y* relationship based on Equation (4), starting from the initial unit-cell dimensions. This diagram clearly shows that as the value of *y* (height) decreases, the value of *x* (width) decreases as well. Using the notations in Figure 9, during compression, the height of the unit cell decreases form $y_{0}$ to $y_{1}$, so the angle value of $\varphi_{0}$ decreases to $\varphi_{1}$. As the angle value decreases, the value of the cosine function increases; thus, the value of $x_{0}$ decreases to $x_{1}$ (2). Furthermore, the value of the offset parameter will also be smaller (6).

The strain of the unit cell in the *x* and *y* directions (5)–(6) are positive, so Poisson’s ratio (7), which is the negative ratio of the transverse strain to the axial strain, is negative.

(5) $$\varepsilon_{x}=\frac{x_{0}-x}{x_{0}}100\%.$$

(6) $$\varepsilon_{y}=\frac{H-y}{H}100\%.$$

(7) $$\mu =-\frac{\varepsilon_{x}}{\varepsilon_{y}}<0.$$

It is concluded that the considered structure is auxetic and has a negative Poisson’s ratio.

#### 2.7.3. Buckling of the Novel Unit Cell

The deformation of the unit cell is not without limits. As can be seen in Figure 9, as the deformation progresses, the edges of length *s* approach the vertical position, which is considered a critical deformation state, as buckling can occur instead of auxetic deformation. Buckling is an unpredictable, unfavourable behaviour for energy absorption. In other words, the analytical approach assumes the existence of two possible behavioural mechanisms depending on the specimen parameters. 

Critical displacement values in the *x* ($x_{m}$) and *y* ($y_{m}$) direction can be calculated from (8) and (9):

(8) $$x_{m}=l\cos\varphi^{*},$$

(9) $$y_{m}=l\sin\varphi^{*}+s.$$

where the critical deg value ($\varphi^{*}$) is as follows:

(10) $$\varphi^{*}=\alpha -90^{0}.$$

It is clear from the equations above that the instant of critical deformation varies depending on the *offset* ($d_{0}$) and *deg* ($\varphi_{0}$) factors. The units with critical deformation form a uniform vertical beam that is under the action of the compression force. The question is whether this beam will buckle. According to Euler’s buckling theory [85,86], the slenderness ratio λ of a beam consisting of n elements is as follows.

(11) $$\lambda =n(2y_{m})\sqrt{\frac{S}{I_{min}}}.$$

where n is the number of unit cells forming the vertical beam, S is the cross section, and $I_{min}$ is the minimal moment of inertia. The minimal moment of inertia is assumed for the position of critical deformation.

(12) S=bw.

(13) $$I_{min}=2\cdot \left(\frac{1}{12}bw^{3}+x_{m}^{2}bw\right).$$

where b is the width of the unit cell, and w is the depth (dimensions are shown in Figure 4d; *b* = 1 mm and *w* = 30 mm). Buckling can be predicted by determining the critical slenderness ratio:

(14) $$\lambda_{g}=\pi \sqrt{\frac{E}{\sigma_{p}}}.$$

where E is the elastic modulus, and $\sigma_{p}$ is the elastic stress value of the material. Considering the material as perfectly elastic, the critical slenderness ratio factor can be calculated based on the values of Figure 5b.

(15) $$\lambda_{g}=\pi \sqrt{\frac{13.55}{6.122}}=5.110.$$

By comparing the slenderness ratio of a given specimen to the critical value, the occurrence of buckling can be determined. That is, if Equation (16) is true, buckling is inevitable.

(16) $$\lambda \geq \lambda_{g}.$$

Buckling occurs due to the compressive force. The critical buckling force can be calculated according to the following relationship:

(17) $$F_{cr}=\frac{\pi^{2}EI_{min}}{(2ny_{m}{)}^{2}}.$$

Substituting (11) into (17), we obtain the following:

(18) $$F_{cr}=\frac{\pi^{2}ES}{\lambda^{2}}.$$

The critical force depends on the slenderness ratio and the area of the cross-section.

#### 2.7.4. Results of the Analytical Approach

The calculated parameters of the specimens for various values of $\varphi_{0}$ and $d_{0}$ (presented in Table 1) are summarized in Table 2. The analytical approach also allows us to predict the behavioral mechanism of the specimens. If Equation (17) is fulfilled, then buckling occurs; otherwise, favourable continuous auxetic behaviours can be expected. 

For specimens 7 to 9, buckling occurred at low strain values (less than 20%), while for the other specimens, buckling occurred only after the initial auxetic deformation. For specimens 2–3, buckling was not expected. The accuracy and relatability of the analytical approximation can be evaluated by comparing it with the experimental test results. The critical force value can be calculated using Equation (18), based on the material properties and specimen dimensions: $F_{cr}=153.7\;N$. It is important to emphasize that the theoretical model only considers lattice-like behaviour and does not consider the contact between edges (i.e., compaction). Based on real test results, the calculated critical strain values will only be considered as buckling indicators up to the point of compaction, as compaction is the limit of lattice-like behaviour.

## 3. Results

Note that the aim of our novel unit cell was to enhance the mechanical properties of the re-entrant honeycomb; hence, first the compression test results and the data that can be extracted from the tests were evaluated. Then, we investigated the deformation behaviour to determine whether, or with what parameter combinations, can buckling be avoided. Lastly, the accuracy of our analytical approximation and established FEM test environment were compared.

### 3.1. Compression Test

**Figure 11:**
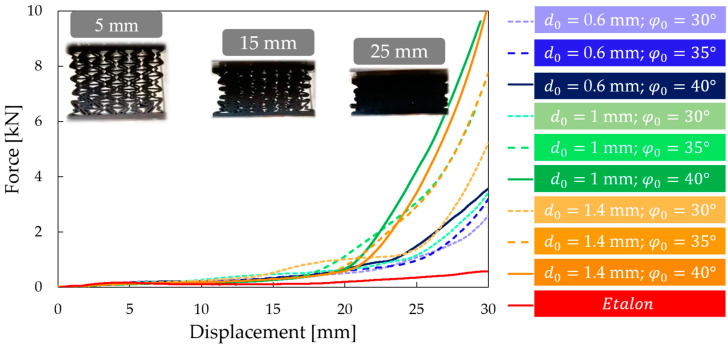
Compression test results; force–displacement curves for all investigated specimens.

Compression tests were performed on all nine previously presented specimens (see Table 1 and Appendix A) as well as on the etalon specimen. Force–displacement curves are shown in Figure 11. 

It can clearly be seen that the maximum compression force was greater for all novel specimens than in the case of the unmodified etalon specimen. The impact of the parameters and geometrical modification were evaluated in a complex analysis.

### 3.2. Description of the Two Typical Behavioural Mechanisms

**Figure 12:**
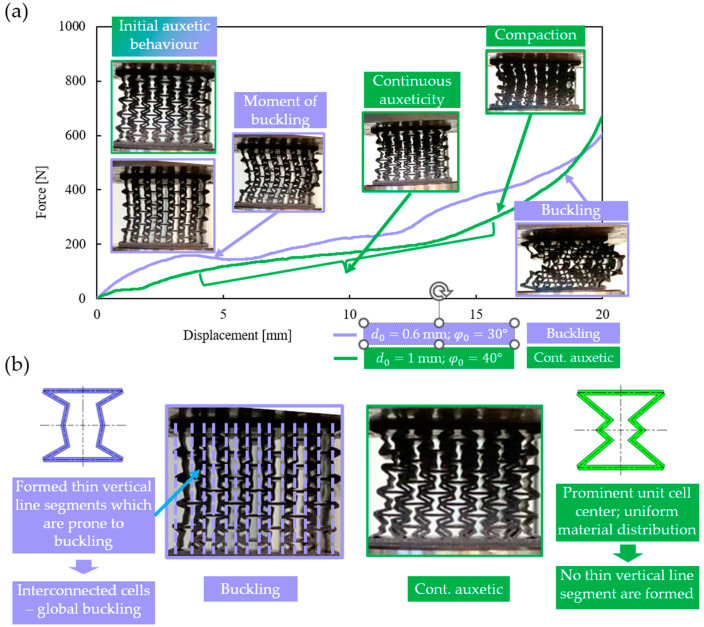
Illustration of the two typical deformation behaviours. (**a**) Comparison of the buckling and continuous auxetic behaviour mechanism in the narrowed 0–20 mm deformation range; (**b**) figures explaining how the initial unit-cell geometry effects the expected deformation behaviour.

Two entirely different behavioural mechanisms were observed during compression testing: a buckling mechanism and a continuous auxetic mechanism (as predicted by the analytical approach). At the initial stages of compression, auxetic behaviour was observed in all cases, but as the deformation progressed, one group of specimens buckled laterally, while others continued to collapse in on themselves, still maintaining the auxetic behaviour. The two different behavioural mechanisms are compared in Figure 12a, up to a deformation of 20 mm. In the narrowed 0–20 mm deformation range, the difference in force–displacement values between the two behaviour mechanisms can easily be observed. 

It can be noted from Figure 12a that both specimens showed auxetic behaviour in the initial stage, until one of them buckled laterally to the right. In case of the specimen with the continuous auxetic behaviour mechanism, no significant buckling could be observed as the compression progressed. After a certain point, the specimen began to compact, which was manifested by a spike in the compression force, whereas no compaction was observed at the same deformation level for the specimen with the buckling mechanism (see Figure 12a).

Deformation behaviour is closely related to the shape of the specimens, and in specimens with certain geometrical parameters, vertical lines are formed at a given level of deformation (Figure 12b). These vertical line segments are still subjected to axial compression; hence, buckling is inevitable. These formed vertical line segments are interconnected by other constituents of the unit cell, resulting in a global buckling of the specimen. It was found that the more prominent the centre of the unit cell (i.e., increasing the *offset* value, resulting in a more uniform material distribution inside the unit cell), the less prone it is to buckling (Figure 12b). At increased offset values, thin vertical segments will not be formed, even at large deformation levels, and the material distribution inside the cell stays uniform; hence, the re-entrant deformation mechanism can behave auxetically at a larger deformation spectrum. This observation can be referred to as a stabilization design guideline for re-entrant auxetic lattices and may have a similar beneficial effect on other lattices (see Section 3.7 as well). 

**Figure 13:**
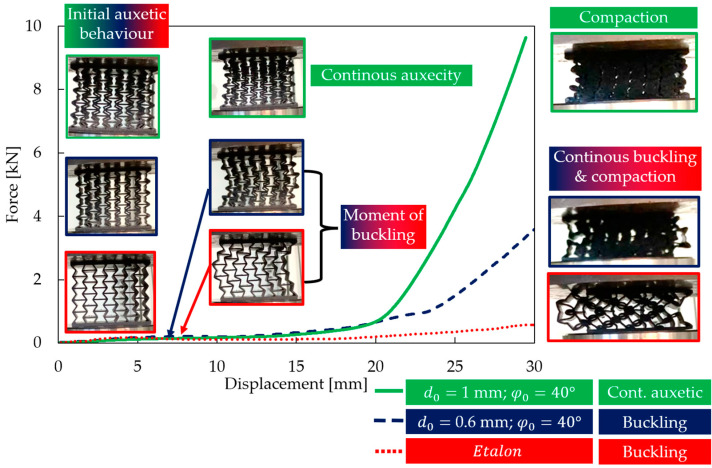
Comparing force–displacement curves of a specimen prone to buckling, a specimen with continuous auxetic behaviour, and the original re-entrant specimen (i.e., etalon).

For a more comprehensive analysis of the two significantly different behavioural mechanisms, the results were also compared over the entire 0–30 mm compression range (Figure 13), supplemented with the results of the auxetic honeycomb (etalon specimen). At the initial, low deformation stage, auxetic behaviour could be observed in all three cases (for all the specimens); however, both etalon and the specimen with the buckling behaviour mechanism bent laterally (buckle) after a certain deformation load. The force–displacement curve of the specimen exhibiting continuous auxeticity tended to have high force values without any regression, while for the other two specimens, the maximum force value was much lower (see Figure 13). At the same time, it can also be observed that the novel geometries, regardless of their deformational behaviour, required greater forces to be compressed. 

### 3.3. FEM Simulation Results

**Figure 14:**
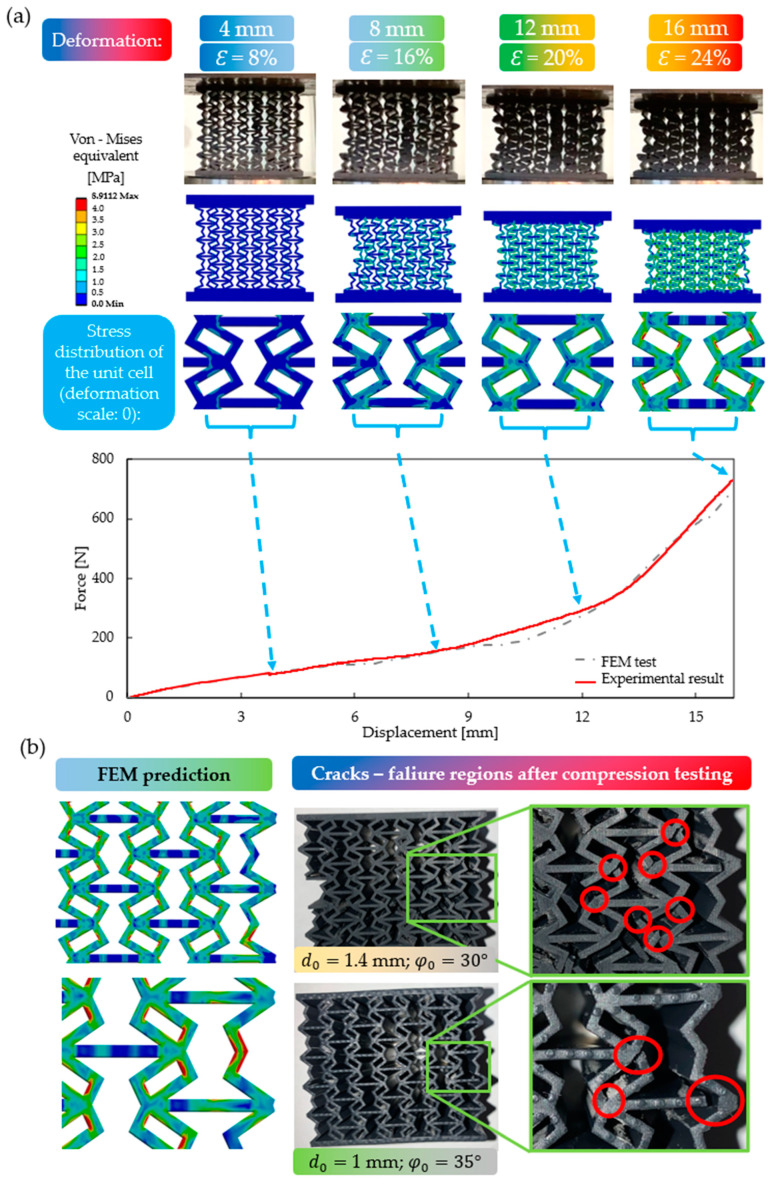
Comparing the real compression test and FEM results of a specimen with continuous auxetic behaviour; the specimen is characterized by the following parameter values: offset = 1.4 mm and deg = 30°. (**a**) Force–displacement values (curves) and deformation images are used for comparing FEM and real test results; (**b**) finite element predictions were used to locate potential cracks (circled in the figure) and failure points in real specimens.

**Figure 15:**
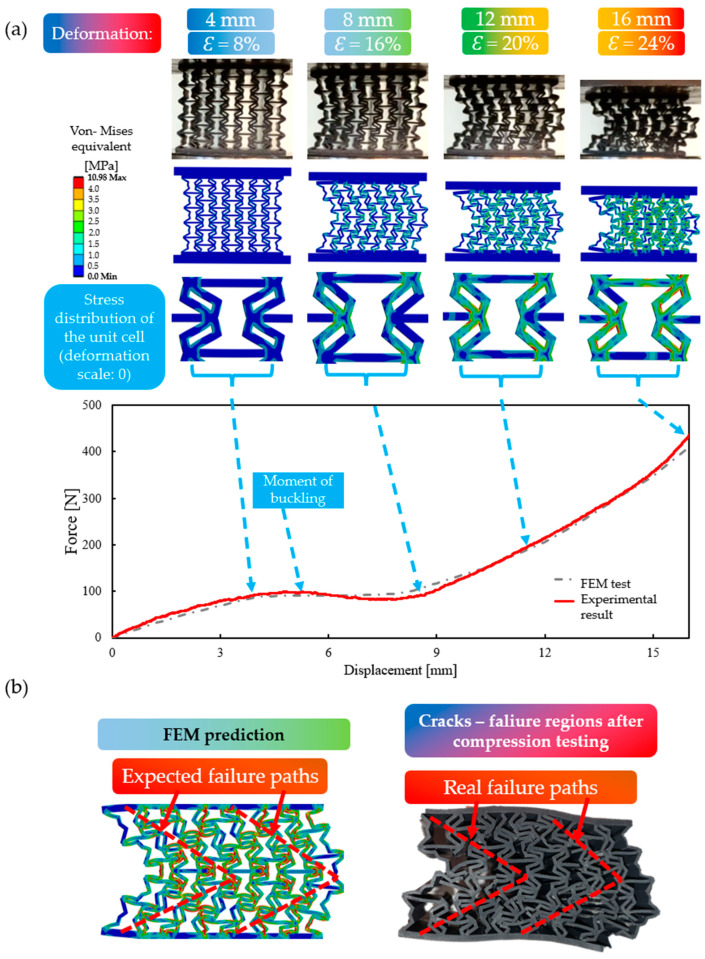
Comparing the real compression test and FEM results of a specimen prone to buckling; the specimen is characterized by the following parameter values: offset = 0.6 mm and deg = 40°. (**a**) Force–displacement values (curves) and deformation images are used for comparing FEM and real test results; (**b**) finite element predictions were used to locate potential cracks and failure paths in real specimens.

Finite element simulation results, as illustrated in Figure 14 and Figure 15, represent real geometric behaviours quite accurately. The two deformation behaviours (buckling and continuous auxetic) are also represented in the finite element simulations; furthermore, the FEM method can also indicate potential failure points by displaying stress distributions. FEM simulations were considered for up to 16 mm (ε≈32%) of deformation, as this can be considered the boundary point of lattice-like behaviour, the point where compaction is not yet overly dominant; cracks do not appear, and unit-cell deformations are still present. FEM results were also considered as a prediction method for crack formations.

#### 3.3.1. Specimens with Continuous Auxetic Behaviour

Figure 14a compares the real and finite element compression test results of a specimen ($d_{0}=1.4\;mm;\;\varphi_{0}=30{}^{\circ}$) with continuous auxetic behaviour along deformation images and force–displacement values. The initial auxetic behaviour of the specimen changed to a slight lateral deflection (Figure 14a—ε=16%), which, within a short time, continued again in continuous auxetic behaviour during further compression.

Figure 14a shows that the force–displacement curves of the FEM and real-life compression test are in good agreement, as are the deformed shapes. The extent of deviations is acceptable, showing the adequacy of the established finite element environment. Since the finite element method can be considered sufficiently accurate, we can also analyse and evaluate the stress distribution obtained and illustrated in Figure 14b. The stress values of 4 MPa or greater are shown in red, which is close to the critical value and may result in possible failures or cracks. In this case, the critical areas are limited to a small region—corner points; otherwise, the stress distribution of the structure can be considered uniform. Cracks and factures that appear in the specimens are in good agreement with the FEM predictions; local damage accumulation was not observed. In light of the above, the continuous auxetic behaviour deformation mechanism is favourable, even in the case of large deformations, where the appearance of cracks is inevitable. 

#### 3.3.2. Specimens with Buckling Behaviour

In the case of specimens prone to buckling, following the initial (Figure 15a ε=0–8%) auxetic behaviour, an increasing lateral buckling could be observed. Similar to the previous section, the deformation images of the experimental and finite element simulations are in good agreement. The deviation between the FEM and experimental results in the initial stages of the force–displacement curves can be explained by the uneven compaction that occurred during only the real-life compression test, which was concentrated at the lower and upper segments (see Figure 15a). 

In the case of specimens with buckling behaviour mechanisms, the stress distribution cannot be considered uniform, even in the early stages of deformation. Lateral buckling guides the line of action of the force, resulting in symmetric oblique regions of high stress concentration (see Figure 15b). The failure traces predicted by the finite element method also appeared during the experiments. The cracks and the uneven, concentrated stress distribution significantly reduce the load-bearing capacity of the specimens, as reflected in Figure 12 and Figure 13. 

The result comparison shows that the buckling behaviour is not only a behaviour mechanism that cannot be planned but also leads to an uneven, concentrated stress distribution, which ultimately affects the compression resistance and thus the energy absorption capacity of the specimen. 

### 3.4. Result Evaluation

The previous sections presented, in detail, the compression test results, the two significantly different behavioural mechanisms, and the applicability and accuracy of the finite element method. However, a numerical evaluation of the results is also required, for which the maximum compressive force and the absorbed energy were chosen, which are common metrics in such tests. 

For the numerical characterization, two points considered to be typical of the specimens were chosen:

- Lattice-like behaviour boundary point: the point where compaction is not yet significant and unit-cell deformations are still present. The results suggest that this point is, on average, at 16 mm of deformation.
- The limit of our measurement range is 30 mm of deformation.

Absorbed energy is defined by the area under the force–displacement curves, as per Equation (19).

(19) $$E_{absorbed}=\int_{0}^{30}f\left(x\right)dx$$

**Figure 16:**
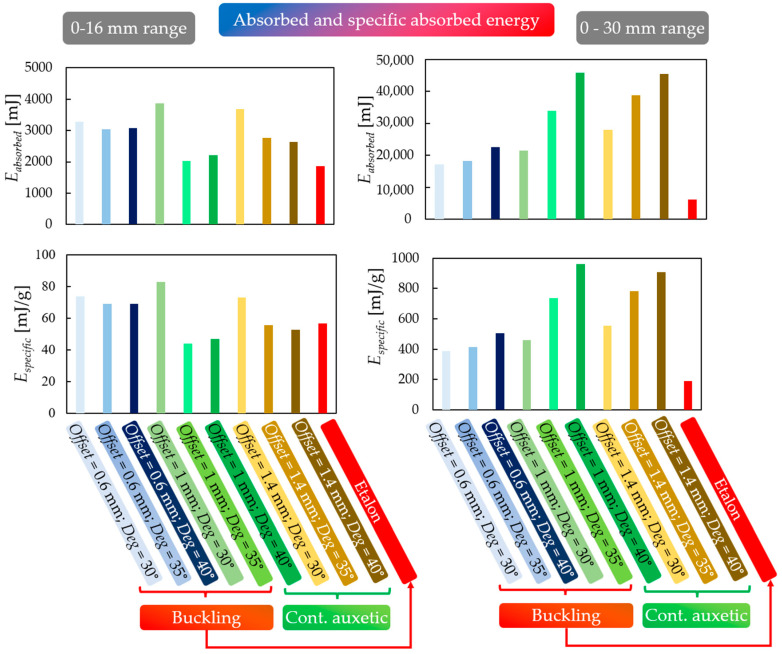
Absorbed and specific absorbed energy of values of all tested specimens in the narrowed 0–16 mm range (i.e., lattice-like behaviour range) and in the extended 0–30 mm deformation range.

Absorbed energy values are shown in Figure 16. In addition to the absorbed energy, the specific energy values are also considered, since the mass of each specimen is different due to varying geometric parameters. 

Figure 16 shows that all tested specimens were capable of higher energy absorption compared to etalon. Considering the mass-specific results, this conclusion is also valid, and it was observed that specimens with continuous auxetic behavioural mechanisms can absorb greater amounts of energy. Specimens with continuous auxetic behaviours are self-compacting as opposed to buckling specimens, which do not densify significantly during compression. This phenomenon explains the difference in the absorbed energy values. However, up to the boundary point of lattice-like behaviour (0–16 mm range), it cannot be said that specimens with continuous auxetic behaviour would be more favourable. Looking at the diagrams, the opposite appears to be the case, which can be explained by comparing the force–displacement curves (Figure 12 and Figure 13). In the initial stage, once buckling occurs in specimens prone to buckling, the compression force increases and takes on higher values than specimens with continuous auxetic behaviours. The deformation resistance of the buckled specimens increases. 

This trend persists until the onset of significant compaction of specimens with continuous auxetic behaviour; at that point, the deformation resistance of these specimens increases and surpasses the ones prone to buckling.

### 3.5. Poisson’s Ratio of Specimens with Continuous Auxetic Behaviour

Based on the results of the finite element method, one can even calculate Poisson’s ratio of the unit cell, whose value changes with the deformation. Poisson’s ratio of a unit cell can be calculated as per Equation (20). The axial and transversal strain of the unit cell is calculated as the quotient of the directional dimension change (∆x and ∆y) and the initial unit-cell dimensions in the axial and transverse directions; see Equations (21) and (22).

(20) $$\mu =-\frac{\varepsilon_{x}}{\varepsilon_{y}}.$$

(21) $$\varepsilon_{x}=\frac{∆x}{W_{Inital}}100\%=\frac{∆x}{2\cdot x_{0}}100\%.$$

(22) $$\varepsilon_{y}=\frac{∆y}{H_{Inital}}100\%=\frac{∆y}{2\cdot H}100\%.$$

**Figure 17:**
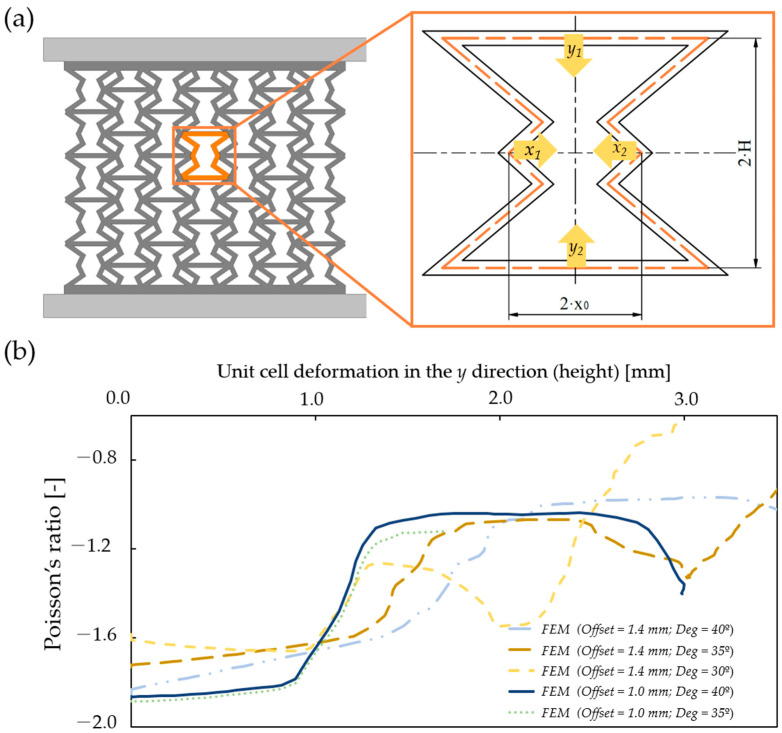
Illustration of directional deformation components used for Poisson’s ratio calculation and the calculated values. (**a**) The four directional deformation components; (**b**) Poisson’s ratio as a function of unit-cell deformation in the y direction for specimens with continuous auxetic behaviour.

To acquire the axial and transversal dimension change of a unit cell, deformation probes were placed in the FEM model, as illustrated in Figure 17a. 

The extracted deformation values formed the basis for determining the directional dimension changes, as per Equation (23):

(23) $$∆x=\frac{x_{1}+x_{2}}{2};∆y=\frac{y_{1}+y_{2}}{2}.$$

Poisson’s ratio was evaluated for all specimens with continuous auxetic behaviour, as illustrated in Figure 17b. The finite element results show that the novel geometry is auxetic; the value of Poisson’s ratio is, on average, −1.7 at low deformations, and as the deformation progresses, its value goes up to around −1.0. One can also observe that the value of Poisson’s ratio depends on the geometrical parameter values, as expected. The curves in Figure 17b appear as if they were shifted from one another, which is explained by the progression of deformation and different initial parameters. As the deformation progresses the initial geometric parameters change and match the parameters of other specimens with different initial parameters; thus, the values of Poisson’s ratio can be considered as “delayed”.

### 3.6. Effect of Geometric Parameters on the Examined Properties and Optimization

**Figure 18:**
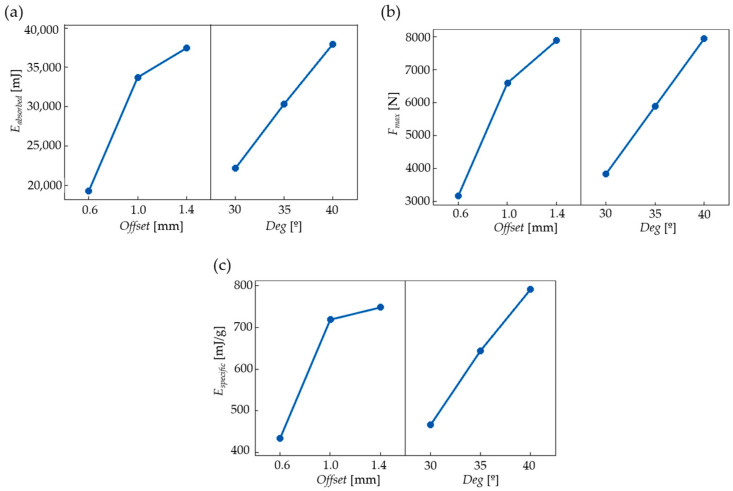
Effects of geometrical parameters (offset and deg) on (**a**) absorbed energy, (**b**) specific energy, and (**c**) maximum compression force on the extended compression range of 0–30 mm.

The main effect plots in Figure 18 describe the effects of the geometrical parameters on the chosen properties (absorbed energy, [mJ]; maximum compression force, [N]; and specific energy, [mJ/g]) for the examined 30 mm compression range. 

According to Figure 18a,c, both the absorbed energy and the maximum force value increases as a result of increasing both geometrical parameters. Figure 18b shows the main effect diagrams of specific energy absorption; similarly, by increasing the parameter values, the absorbed specific energy increases as well.

A mathematical relationship between the geometrical parameters (*deg* and *offset*) and the investigated characteristics can be sought using phenomenological models. The output parameter (*Z*) can be interpreted as a function of two independent variables (*X* and *Y*) (see Equation (24)):

(24) Z=f(X,Y).

where *X* and *Y* are input-independent parameters (in our case, *X* is the *offset* [mm], and *Y* is the *deg* [°]). *Z* is the predicted output parameter (in our case, it is absorbed energy, [mJ]; maximum compression force, [N]; and specific energy, [mJ/g]). The equation that can describe a second-order [87] trend surface is shown in Equation (25):

(25) $$Z=a_{0}+a_{1}\cdot X+a_{2}\cdot Y+a_{3}\cdot X^{2}+a_{4}\cdot Y^{2}+a_{5}\cdot XY+\varepsilon .$$

where $a_{0},a_{1},a_{2},a_{3},a_{4},a_{5}$ are constant coefficients that are estimated with the help of the available data, and *ε* is the error of the model [88,89]. The equations, based on (25), are as follows:

(26) $$\begin{array}{cc} E_{absorbed}= & -37416+36312\cdot Offset+814\cdot Deg-33491\cdot {Offset}^{2}\\ & \;-11\cdot {Deg}^{2}+1525\cdot Offset\cdot Deg\\ & R^{2}=0.9418, \end{array}$$

(27) $$\begin{array}{cc} F_{max}= & -2269+1126\cdot Offset-104\cdot Deg-6671\cdot {Offset}^{2}+517\\ & \;\cdot Offset\cdot Deg\\ & R^{2}=0.9493, \end{array}$$

(28) $$\begin{array}{cc} E_{specific}= & -1334+962\cdot Offset+47\cdot Deg-797\cdot {Offset}^{2}-0.62\\ & \;\cdot {Deg}^{2}+29.3\cdot Offset\cdot Deg\\ & R^{2}=0.9305, \end{array}$$

The phenomenological models presented above can be qualified by their residuals, i.e., the difference between the estimated and measured values. The models (Equations (26)–(28)) are found to have residuals that are random, follow a nearly normal distribution, and have expected values that are practically zero (Appendix C). This proves that the equations above describe the relationship between the geometric parameters and the properties under investigation quite well. 

**Figure 19:**
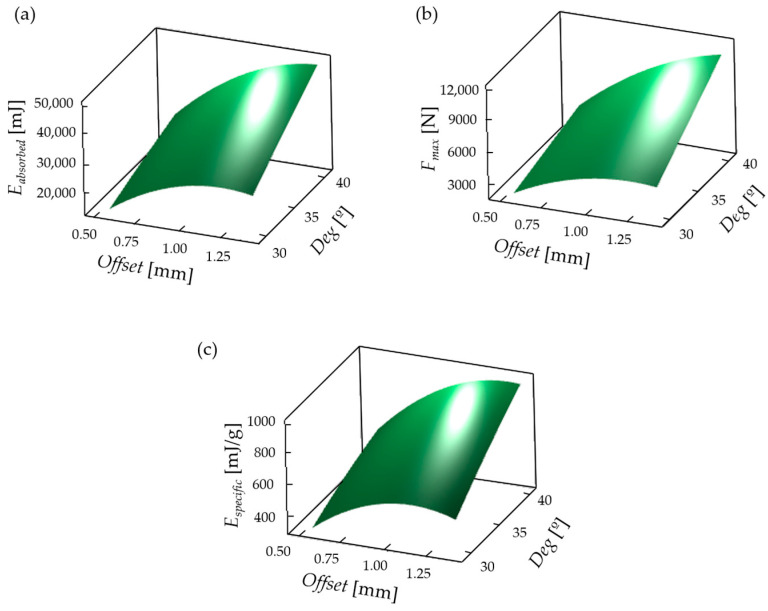
Effects of geometric parameters on (**a**) absorbed energy, (**b**) maximum force, and (**c**) specific energy in 3-dimensional graphical representations (at the studied 0–30 mm compression range).

The graphical representation of Equations (26) and (28) (Figure 19) similarly describes the effects of the geometric parameters on the absorbed energy, the specific absorbed energy, and the maximum force values. As can be seen from the plots of Figure 19, all studied characteristics increased with increasing parameter values; however, the specific energy (Figure 19c) has a maximum value at the studied range. Consequently, it is worth looking for an optimum in the studied parameter range, where the specific energy is maximized, in addition to the maximum energy absorbed and the maximum compression force. 

#### Optimization

The three objective functions in search of the optimum are as follows:

(29) Absorbed energy→MAX,

(30) Specific absorbed energy→MAX,

(31) Compressive force→MAX.

The optimum of the three objective functions (Equations (29) and (31)) can be determined by the so-called desirability functions [90]. These desirability functions can take values from the interval (0–1). The higher the disability value, the closer we are to the achievable optimum. In our case, the desirability functions are $d_{Eabsorbed},\;d_{Fmax},$ and $d_{specific\;energy}$ (see Appendix D). The bounds of these functions are set to correspond to the minimum and maximum values measured in the experimental tests. The objective functions are shown in Appendix D. The optimum value can be determined from the so-called composite desirability function (*D*), whose maximum value is the optimum. The composite desirability function can be calculated as follows:

(32) $$D=\sqrt[{3}]{d_{Eabsorbed}\cdot d_{Fmax}\cdot d_{Especific}}$$

As a result of the optimisation method described above, the optimal values (over the studied parameter range) are deg=40° and Offset=1.33 mm. These optimal parameters are expected to yield $E_{absorbed}=47933\;mJ$, $F_{max}=10741\;N$, and $E_{specific}=967.5\;mJ/g$.

**Figure 20:**
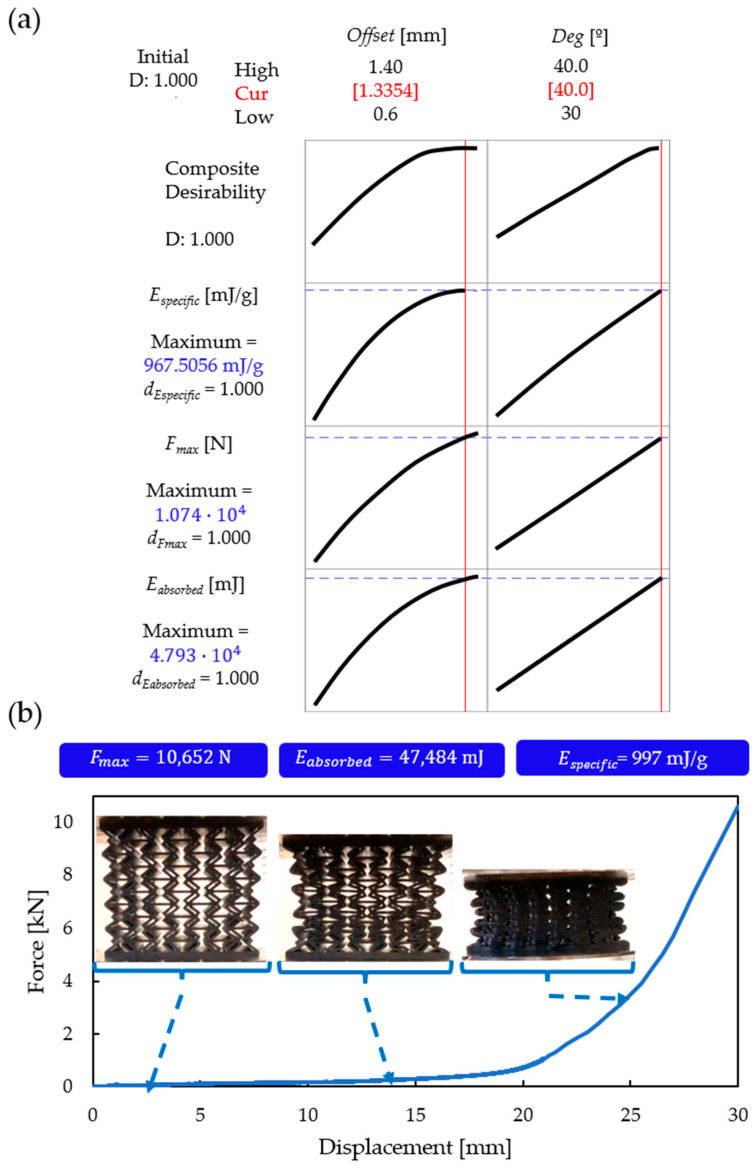
The results and the validation of the optimizer. (**a**) Results of the optimizer; (**b**) compression test results of the optimal specimen, characterized by the following parameter values: offset = 1.33 mm and deg = 40°.

Based on the determined optimum parameters (Figure 20a), a specimen was printed for compression testing. The compression test results are shown in Figure 20b, alongside the maximum force, absorbed energy, and specific absorbed energy values. The experimental results are in good agreement with the predictions of the optimisation method; furthermore, the specimen exhibited continuous auxetic behaviour. It is concluded that the novel unit-cell geometry has an optimal design. 

### 3.7. Behavioural Mechanisms and Parameter Effects—Comparing the Results with the Analytical Prediction

The analytical approach (Section 2.7) predicted the existence and parameter dependence of the two behavioural mechanisms. This phenomenon also recurred in the experimental compression tests and finite element studies. Table 3 summarises the relationship between behavioural mechanisms and parameters and compares the results of the analytical approximation with the experimental test results. The analytically determined critical strain values were only considered as indicative of buckling till compaction (ε≈32%) (i.e., in the lattice-like behaviour range) (see Section 2.7.4 and Section 3.3). The finite element studies reflected the experimentally observed mechanism in all cases.

Table 3 shows that this analytical approach can be considered sufficiently accurate. For specimen numbers 3 to 5, the analytical method predicted a relatively high critical strain value at which buckling would occur; at this range, the applied approximations (the α angle was assumed to be constant, and the individual deformation and contact of the edges were not considered) explain the deviation from the experimental results. Also, based on the previously presented results (Section 3.3.1, Figure 14a), minimal lateral buckling could be observed in specimens with continuous auxetic behaviour. 

**Figure 21:**
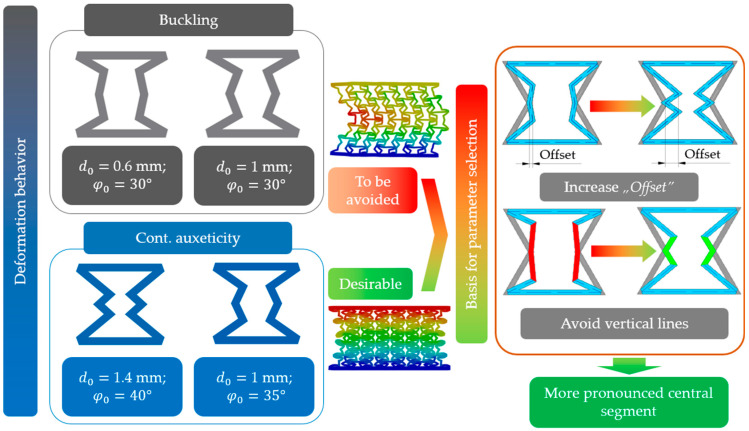
Design guidelines with examples for achieving high-performance continuous auxetic behaviours in the novel unit cell.

Furthermore, based on the results of Table 3, it can be concluded that increasing the “*offset*” parameter ensures continuous auxetic behaviour, thus avoiding the occurrence of buckling and resulting in a much stiffer structure. Directing our attention to the middle segment of this table (*offset* = 1 mm), we can see that for the same offset parameter, a different behaviour is observed as a function of the “*deg*” parameter; that is, the “offset” parameter is not the only determinant of the behaviour mechanism. At the same time, this table clearly shows the boundary ($d_{0}=1\;mm;\;\varphi_{0}=35{}^{\circ}$) between the behaviour mechanisms. Our aim was to investigate the root of this phenomenon to obtain high-performance, predictable behaviours, thus specimens with continuous auxetic behaviours. By carefully examining the unit-cell geometries, the desired behaviour can be achieved by following the design guidelines shown in Figure 21. 

Based on Figure 21, buckling can be avoided by implementing two geometry modifications. One can see from Table 3 that with a significant increase in the offset parameter, buckling can be avoided regardless of the deg parameter. The second necessary geometry modification is the avoidance of vertical lines, and long vertical segments under compression are prone to buckling (as detailed in Section 2.7.3). Figure 21 illustrates the correct and incorrect layouts by means of examples. Summarising the two necessary geometry modifications mentioned above, the creation of a more pronounced central unit-cell segment is required. These stabilization design guidelines are also supported by the analytical approach. Reviewing the results in Table 3, one can see that the critical strain value decreases with decreasing “*offset*” parameter values and with parameter combinations that result in a less pronounced central segment. The new breakpoints of the novel, doubly re-entrant structure allowed for the creation of a more centrally pronounced unit-cell design. Furthermore, the added breakpoints added additional degrees of freedom. The additional deformation freedom with the proper parameter selection can result in high performance, non-buckling behaviours. 

This section showed that the analytical description was able to predict the expected behavioural mechanism with sufficient accuracy. Using the same analytical approach, the critical force value $F_{cr}=153.7\;N$ was also calculated, which indicates the initiation of buckling for specimens susceptible to it. 

**Figure 22:**
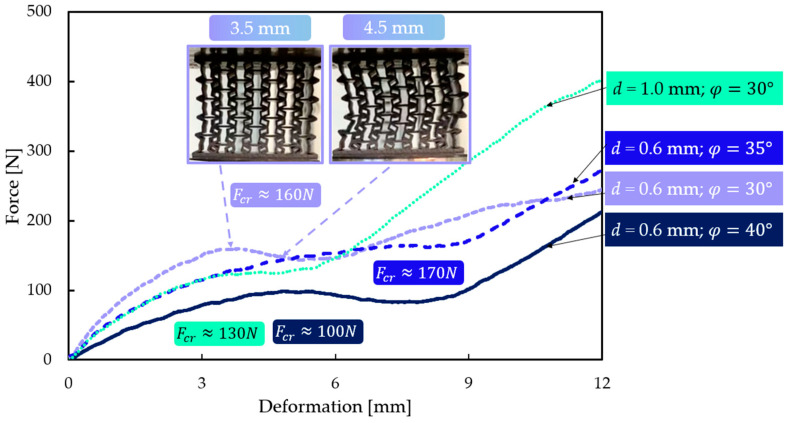
Force–displacement curves of specimens prone to buckling; buckling is observed as a regression in force values. These regression points are highlighted with force values.

Figure 22 shows the experimental force–displacement curves of specimens susceptible to buckling at the moment of buckling. Figure 22 also illustrates the presence of auxetic behaviour until the critical force is reached, at which point buckling ensues immediately. The sudden change in behaviour can also be well traced by a regression in the force–displacement curve for each specimen. The mean critical force value is 140 N, which is approximated, quite accurately, by the calculated $F_{cr}=153.7\;N$ force value. Critical buckling force values were also extracted from our FEM simulations and are listed in Table 4.

The average FEM buckling force is 132.9 Newtons, which is in good agreement with the analytical approximation and real compression test results.

#### Specimen Stiffness

Specimen stiffness was also considered and calculated as the quotient of the von Mises stress and strain for all specimens and was compared with the original (etalon) specimen (see Table 5). The specific stiffness was obtained by dividing the stiffness values with the corresponding density values in Table 1. The stiffness of the novel design is lower than that of etalon. Higher stiffness values were observed for specimens prone to buckling, such as etalon. Only the *offset* parameter effected the specimen’s stiffness, and the *deg* parameter had a negligible impact on stiffness.

## 4. Discussion

Improving the stability and properties of the re-entrant honeycomb structure has attracted the interest of many fellow researchers. Ramen et al. [65] studied a series of bio-inspired modifications, including the similar-looking stiff re-entrant structure with a horizontal road connecting the original breakpoints of the re-entrant structure. In their study, the additional horizontal road segment limited the in-cell deformation, and, as a result, the structure did not retain its auxetic behaviour. Furthermore, the authors did not consider the neighbouring effect, as unit cells were not organized as a regular lattice. The novel, doubly re-entrant design presented in this article was studied as a regular lattice; thus, the neighbouring effect was also considered. Furthermore, owing to the different geometrical design where no continuous horizontal edges are formed (as would be the case if the structure presented in the work of Ramen et al. [65] was latticed), the in-cell deformation in unlimited, auxetic behaviours was retained. The unit-cell design investigated by Ramen et al. [65] is advantageous as far as deformation is concerned, as buckling is less evident but still occurs, as shown by a different study [64]; hence, it would be of interest to study this novel unit-cell design in lattice patterns.

In our paper, the doubly-entrant structure was achieved by geometrical modification (with the introduction of breakpoints; Section 2.1). A remotely similar-looking unsymmetrical structure was studied by Lantada et al. [38] for the development of resonant structures. These authors based their design on a chiral structure, showing that similar structures can be achieved by different methods. Our aim was to comprehensively study this novel, doubly re-entrant structure; hence, the design is based on a geometrical modification and is characterized by the parameters that form the basis of these modifications. Fellow research works also lack the effects of geometrical parameters. In this paper, with the two geometrical parameters (*offset* and *deg*), we were able to demonstrate that the doubly re-entrant structure can exhibit varying deformation behaviours and a spectrum of mechanical parameters. Owing to parameter-based studies, our study showed that buckling can be avoided, which is characteristic of re-entrant structures. Single specimen-based studies may miss certain attainable properties.

Improved mechanical properties make the doubly re-entrant structure applicable in all eras where the original re-entrant honeycomb structure is applied or would be applied, but its mechanical properties are insufficient. An improved deformation stability, indentation resistance, and energy absorption capacity makes the doubly re-entrant structure applicable in sport helmets, knee pads, and in fall-impact protectors. Since this novel structure can still be considered a simple design, it can also be used in high-stress eras (such as ship constructions [91]) using traditional manufacturing technologies.

Our article has looked at the novel, doubly re-entrant structure in an outstandingly broad way. The parameter dependency of mechanical parameters and behaviour mechanisms are now understood. Furthermore, owing to the broad study range, the formulated design guidelines can advance the design of several novel lattices. A regular lattice arrangement allowed us to understand the structure as it may be implemented in real-life applications. It may be worth considering the effects of a triple or quadruple re-entrant unit-cell design in future work, although increasing the number of breakpoints above a certain threshold might reduce the stability. The presented design can, in the future, be extended into three dimensions as well, as did Wang et al. [92] in their work. It would also be of interest to further improve the deformation behaviour of this novel structure, making the deformation behaviour parameter independent, so that only mechanical properties would change with the parameters.

## 5. Conclusions

In this study, a novel, doubly re-entrant unit cell was proposed to improve the properties and eliminate the disadvantages of the widely used auxetic honeycomb. The geometrical modification was characterized by two parameters (*offset* and *deg*). The effects of the geometrical modification were evaluated across a wide parameter spectrum. Not only the mechanical parameters but also the deformation behaviours of the specimens formed the scope of our study. Specimens were subjected to compression tests in real-life and in a FEM environment as well. Furthermore, analytical approximation was used to predict the expected behaviour. Specimens were prepared from a unique material mix, which allowed us to study the deformation behaviour even at large deformations. Based on our investigation, the following conclusions can be drawn:

- Owing to the wide spectrum of the two parameters (offset: 0.6 … 1.4 mm; deg: 30° … 40°), their effects can be tested extensively.
- The proposed novel geometry shows improved mechanical properties compared to the auxetic honeycomb structure, regardless of the parameter values.
- Increasing the *offset* and *deg* parameters results in an increased energy absorption capability and maximum compressive force.
- The specific energy absorption capability of the novel specimens increases with increasing geometrical parameters (*offset* and *deg*).
- Based on an optimisation method using desirability functions, the optimal geometric parameters in the considered parameter range are *deg*, 40° and *offset*, 1.33 mm. The expected properties and auxeticity of the optimal specimens were also verified with experimental compression tests.
- The specimens exhibited two distinctly different behavioural mechanisms, namely, buckling and continuous auxetic behaviours.
- At low deformations, specimens with buckling behaviour showed better mechanical properties; however, buckling is an unpredictable behaviour mechanism.
- At high deformation loads, specimens with continuous auxetic behaviour showed more favourable mechanical properties.
- Following the proposed stabilization design guidelines, the expected operating mechanism can be designed while achieving more favourable mechanical properties as well.
- The established finite element environment is a sufficiently accurate representation of the real measurement results and the expected behaviour mechanism.

## Figures and Tables

**Table 1:** Initial parameters of unit cell.

Specimen No.	1	2	3	4	5	6	7	8	9
*d*_0_ (mm)	0.6	0.6	0.6	1	1	1	1.4	1.4	1.4
$\varphi_{0}{(}^{0})$	30	35	40	30	35	40	30	35	40
Relative density (-)	0.416	0.413	0.409	0.436	0.433	0.431	0.458	0.456	0.457
$\mathrm{Density}\;(kg/m^{3}$)	457.8	454.5	449.9	479.4	475.9	473.7	503.6	502.1	503.3

**Table 2:** Calculated parameters of the structure for various values of $\varphi_{0}$ and $d_{0}$.

Specimen No.	1	2	3	4	5	6	7	8	9
$\varphi_{0}$ (°) $d_{0}$ (mm)	30 1.4	35 1.4	40 1.4	30 1	35 1	40 1	30 0.6	35 0.6	40 0.6
l (mm)	4.792	5.066	5.417	4.330	4.578	4.895	3.868	4.089	4.373
s (mm)	2.743	2.326	1.898	2.784	2.360	1.901	2.892	2.491	2.042
α (°)	89.31	87.99	82.47	98.94	99.93	98.26	108.1	111.06	112.9
$x_{m}$ (mm)	0.708	0.437	0.129	1.223	0.985	0.656	1.798	1.684	1.471
$y_{m}$ (mm)	2.692	2.149	1.188	3.461	3.117	2.849	4.089	3.960	3.744
$\varepsilon_{y\;krit}$	43.40	54.82	75.02	27.24	34.46	40.11	14.05	16.75	21.29
λ	6.197	4.957	2.744	7.914	7.154	6.561	9.245	8.978	8.525
Expected behavioural mechanism	Buckling	Cont. aux	Cont. aux	Buckling	Buckling	Buckling	Buckling	Buckling	Buckling

**Table 3:** Relationship between geometrical parameters and behavioural mechanisms.

No.	Offset Parameter $d_{0}$ [mm]	Deg Parameter $\varphi_{0}\;[{}^{\circ}]$	Analytical Prediction		Real (and FEM)
			Behavioural Mechanism	Critical Strain Value	Behavioural Mechanism
1	1.4	40	cont. auxetic	75.1%	cont. auxetic
2	1.4	35	cont. auxetic	54.8%	cont. auxetic
3	1.4	30	buckling	43.4%	cont. auxetic
4	1	40	buckling	40.1%	cont. auxetic
5	1	35	buckling	34.5%	cont. auxetic
6	1	30	buckling	27.2%	buckling
7	0.6	40	buckling	21.3%	buckling
8	0.6	35	buckling	16.8%	buckling
9	0.6	30	buckling	14.1%	buckling
10	Etalon	0			buckling

**Table 4:** FEM critical buckling force values.

Specimen No.	1	7	8	9
$\varphi_{0}$ (°) $d_{0}$ (mm)	30 1.0	30 0.6	35 0.6	40 0.6
Critical buckling force [N]	139.4	151.6	123.4	117.4

**Table 5:** Specimen stiffness.

Specimen No.	ETA	1	2	3	4	5	6	7	8	9
*d_0_* (mm)	0	0.6	0.6	0.6	1	1	1	1.4	1.4	1.4
$\varphi_{0}\;({}^{\circ})$	0	30	35	40	30	35	40	30	35	40
Relative stiffness (kNm/kg)	43.49	28.88	32.00	31.01	27.75	27.64	27.83	26.10	26.63	26.41

**Figure A1:**
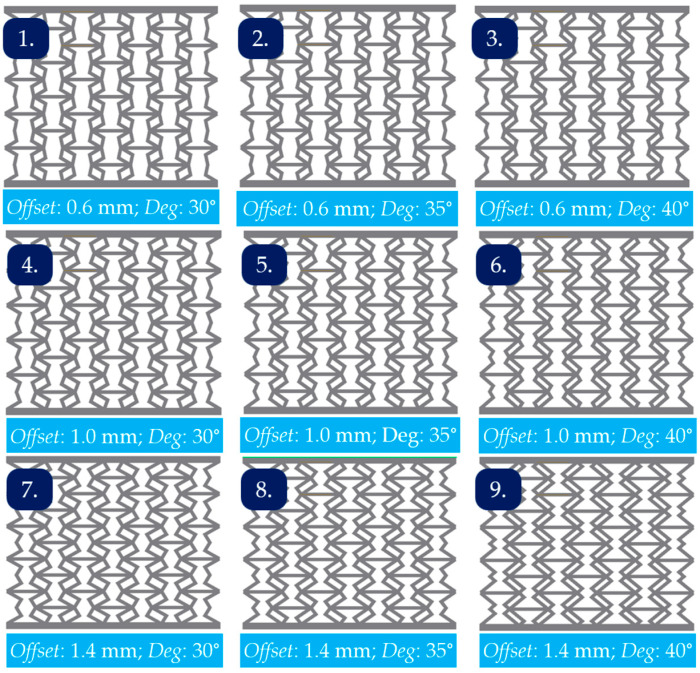
CAD models of the specimens to be tested based on the initial parameters listed in Table 1.

**Figure A2:**
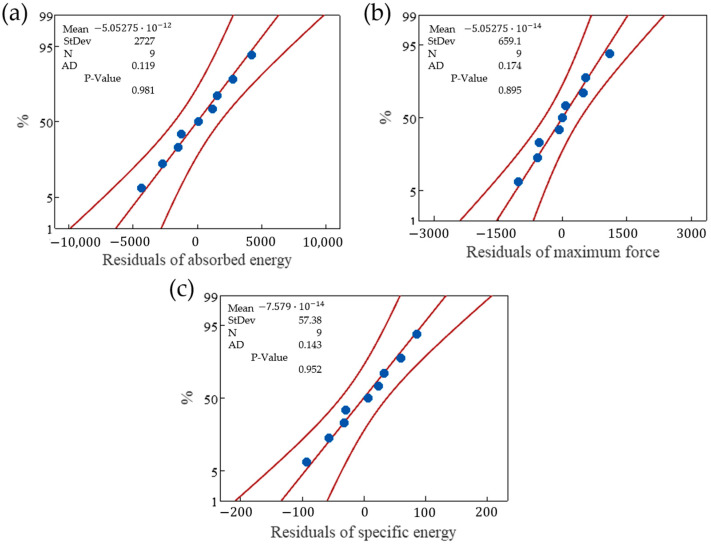
The errors of (**a**) Equation (27), (**b**) Equation (28), and (**c**) Equation (29) on a probability plot.

**Figure A3:**
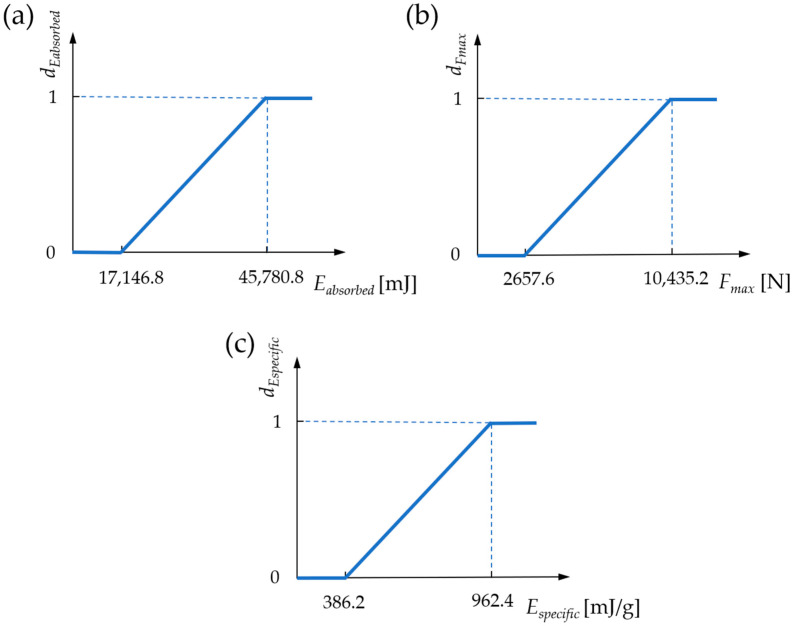
Desirability functions used for the optimization. (**a**) Desirability function of the absorbed energy; (**b**) desirability function of the maximum compressive force; (**c**) desirability function of the specific absorbed energy.

## Data Availability

The original contributions presented in the study are included in the article, further inquiries can be directed to the corresponding author.

## Nomenclature

$d_{0}$	[mm]	Initial *offset* value of the novel specimen
$\varphi_{0}$	[°]	Initial *deg* value of the novel specimen
t	[mm]	Re-entrant edge length of the auxetic honeycomb unit cell
L	[mm]	Half width of the unit cell
H	[mm]	Half height of the unit cell
*ψ*	[°]	Re-entrant angle
l	[mm]	Upper re-entrant edge length of the novel unit cell
s	[mm]	Lower re-entrant edge length of the novel unit cell
$x_{0}$	[mm]	Initial half central width of the unit cell
w	[mm]	Depth of the specimen
b	[mm]	Unit-cell cross-section characteristic thickness
α	[°]	The angle enclosed by the edges of length l and s
y	[mm]	Height of the unit cell (its value changes with deformation)
x	[mm]	Width of the unit cell (its value changes with deformation)
d	[mm]	*Offset* of the novel unit cell (its value changes with deformation)
φ	[°]	*Deg* of the novel unit cell (its value changes with deformation)
$\varepsilon_{x}$	[%]	Axial strain
$\varepsilon_{y}$	[%]	Transverse strain
μ	[-]	Poisson’s ratio
$x_{m}$	[mm]	Critical displacement value in x direction (width)
$y_{m}$	[mm]	Critical displacement value in y direction (height)
$\varphi^{*}$	[°]	Value of φ at the critical moment
λ	[-]	Slenderness ratio
n	[-]	Number of unit cells forming the vertical beam
S	[${mm}^{2}$]	Cross-section
$I_{min}$	[${mm}^{4}$]	Minimal moment of inertia
$\lambda_{g}$	[-]	Critical slenderness ratio
E	[MPa]	Elastic modulus of the material
$\sigma_{p}$	[MPa]	Elastic stress value of the material
$F_{cr}$	[N]	Critical buckling force
$E_{absorbed}$	[mJ]	Energy absorbed by specimens
$E_{specifc}$	[mJ/g]	Specific absorbed energy of specimens
$F_{max}$	[N]	Maximum compression force
X,Y	[-]	Input parameter for the phenomenological model
Z	[-]	Predicted output for the phenomenological model
$d_{Eabsorbed}$	[-]	Desirability function of absorbed energy
$d_{Fmax}$	[-]	Desirability function of maximal compressive force
$d_{specific\;energy}$	[-]	Desirability function of specific energy
D	[-]	Composite desirability function
∆x	[mm]	Dimension change in the x direction
∆y	[mm]	Dimension change in the y direction
$W_{Inital}$	[mm]	Initial unit-cell width
$H_{Inital}$	[mm]	Initial unit-cell height
*a*_0_, *a*_1_, *a*_2_, *a*_3_, *a*_4_, *a*_5_	[-]	Constant coefficients of predicted models
*ε*	[-]	Error of the predicted models

## Appendix A

### CAD Models of Specimens

CAD model of specimens and representations of each parameter combination:

## Appendix B

### Unit-Cell-Geometry Equations

The unit-cell geometry can be described by the following equations:

(A1) $$H\equiv t\sin\psi =l\sin\varphi_{0}+\sqrt{s^{2}-d_{0}^{2}},$$

(A2) $$x_{0}\equiv L-t\cos\psi =L-l\cos\varphi_{0}+d_{0}.$$

where l and s are unknown values. Their length depends on the initial *offset* $d_{0}$ and *deg* $\varphi_{0}$ parameters. Their value is given by the following equations:

(A3) $$l=\frac{L-x_{0}+d_{0}}{cos\varphi_{0}},$$

(A4) $$s=\sqrt{(H-\left(L-x_{0}+d_{0}\right)tan\varphi_{0}{)}^{2}+d_{0}^{2}}.$$

Based on the given (constant) geometric dimensions indicated in Figure 8, the given H and $x_{0}$ dimensions can be determined using Equations (A1) and (A2). The calculated values are H=4.763 mm and $x_{0}=2.75\;mm$. Based on these dimensions, the values of l and s dimension are summarized in Table 1 as a function of the chosen parameter combinations.

**Table A1:** The l and s dimensions of the novel unit cell based on the initial *offset*, $d_{0},$ and *deg*, $\varphi_{0}$, parameter values.

Specimen No. (from Figure A1)	1	2	3	4	5	6	7	8	9
$\varphi_{0}$ (°) $d_{0}$ (mm)	30 1.4	35 1.4	40 1.4	30 1	35 1	40 1	30 0.6	35 0.6	40 0.6
*l* (mm)	4.792	5.066	5.417	4.330	4.578	4.895	3.868	4.089	4.373
*s* (mm)	2.750	2.326	1.898	2.784	2.360	1.901	2.892	2.491	2.042

The analytical description can be simplified, since the initial offset, $d_{0}$, parameter can be expressed as a function of the deg, $\varphi_{0},$ parameter as follows:

(A5) $$d_{0}=scos(\alpha -\varphi_{0}).$$

where α is the angle enclosed by the edges of length l and s, also marked in Figure 8 and Figure 9. This angle is assumed to be constant during the deformation in the analytical approach.

(A6) $$cos\alpha =\frac{l^{2}+s^{2}-t^{2}}{2ls}.$$
